# Peri-oral Monkeypox Virus Infection: A Clinical Report with Confirmatory Polymerase Chain Reaction Findings

**DOI:** 10.3390/vaccines11010036

**Published:** 2022-12-23

**Authors:** Francesca Ambrogio, Carmelo Laface, Anna Paola De Caro, Daniela Loconsole, Francesca Centrone, Teresa Lettini, Gerardo Cazzato, Domenico Bonamonte, Caterina Foti, Maria Chironna, Paolo Romita

**Affiliations:** 1 Section of Dermatology and Venereology, Department of Biomedical Sciences and Human Oncology (DIMO), University of Bari “Aldo Moro”, 70124 Bari, Italy; 2 Interventional and Medical Oncology Unit, IRCCS Istituto Tumori “Giovanni Paolo II”, Viale Orazio Flacco 65, 70124 Bari, Italy; 3 Department of Biomedical Science and Human Oncology (DIMO), University of Bari “Aldo Moro”, 70124 Bari, Italy; 4 Section of Pathology, Department of Emergency and Organ Transplantation (DETO), University of Bari “Aldo Moro”, 70124 Bari, Italy

**Keywords:** Mpox virus, MPXV, clinical features, skin lesions, MSM, infectious disease

## Abstract

Mpox Virus (MPXV) is a zoonotic infectious disease first identified in 1970 in rural villages in rainforest areas of central and western Africa when smallpox was in the final stages of eradication. Since May 2022, cases and sustained transmission chains of monkeypox have been reported for the first time in countries where the disease is not endemic and without cases having direct or immediate epidemiological links to areas of West or Central Africa (travel, importation of mammals). On 23 July 2022, WHO declared monkeypox a “Public Emergency of International Concern” (PHEIC). In this paper, we report two cases of a Polymerase Chain Reaction (PCR)-confirmed MPXV infection. A 39-year-old Italian male came to our attention for a suspected herpetic infection, fever, headache, and malaise, which were followed by the development of an erythematous plaque covered by vesicles on the chin, an oval ulcer with a white peripheral border on the lower lip, and a central erosive area and three pustules on the arms and trunk. During the physical examination, cervical lymphadenopathy was also detected. PCR investigation of the patient and his partner confirmed the presence of MPXV infection. Our report describes a possible clinical feature of Mpox disease and illustrates the challenge of a disease that seems to present itself in different ways.

## 1. Introduction

Mpox virus (MPXV) is a member of the Orthopoxvirus group, related to the smallpox virus, and characterized by the presence of two distinct clades (or strains): the West African strain (Clade two (II)) and the Congo Basin strain (Clade one (I)) [1]. Although, historically, this human disease was mainly limited to sporadic cases and occasional outbreaks (mainly but not exclusively in Africa) [2], as of May 2022, cases of MPXV have been reported in approximately 70 countries where the disease was not considered endemic [1,2]. This outbreak has led the World Health Organization (WHO) to declare the MPXV outbreak “a public health emergency of international concern” [3]. The literature reports that almost all cases associated with the 2022 epidemic are caused by the West African strain and, in epidemiological terms, have been recorded mainly in “Men who have Sex with Men”

(MSM) [1,2,3]. In the case of animal-to-human transmission, MPXV is likely transmitted through body fluids, including saliva or respiratory droplets, and direct contact with exudate from the skin lesion [4]. Instead, person-to-person transmission occurs through close prolonged contact, with the main routes of transmission being respiratory droplets, direct contact with infectious lesions and/or other body fluids, and contact with fomites (contaminated, for example) [5]. On the other hand, it is important to remember the possibility of the transmission human-to-animal (as recently observed in SARS-CoV-2 infection) because it is important to understand the true impact of this “inverse” modality of disease transmission [4]. The recognition of MPXV skin lesions is an essential step in allowing the clinician to suspect this infection (also out of an endemic context) and make an adequate diagnosis. Clinically, the patient presents a rash that occurs within one-three days of the onset of fever, typically starting on the face (involved in 95% of cases) and then spreading to other parts of the body, especially the extremities (including the palms and the soles of the feet (in 75% of cases). The oral mucous membranes (in 70% of cases), the genitals (in 30% of cases), and the conjunctivas (20%) may also be involved. Eye involvement can lead to corneal ulcers and blindness. The rash generally progresses in sequence from macules (lesions with a flat base) to papules (slightly raised firm lesions), vesicles (lesions filled with clear fluid), pustules (lesions filled with yellowish fluid), and crusts that dry and fall off. The number of injuries ranges from a few to several thousand. Unlike chickenpox, the lesions are usually the same size, and the maturational stage happens by anatomical site [5,6]. In this paper, we present a case report of a Polymerase Chain Reaction (PCR)-confirmed MPXV infection, detail the typical skin lesions associated with the disease, and discuss our findings in light of the latest research.

## 2. Case presentation

**Figure 1:**
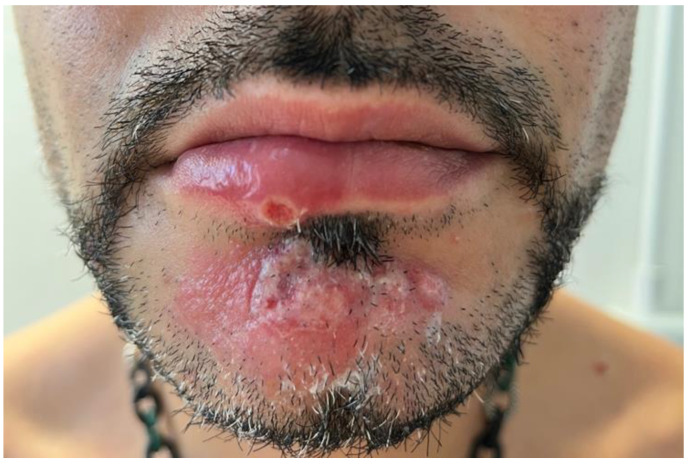
The clinical picture of patient 1: note an erythematous plaque covered in vesicles on the chin, an oval ulcer on the lower lip with a white peripheral border, and a central erosive area.

**Figure 2:**
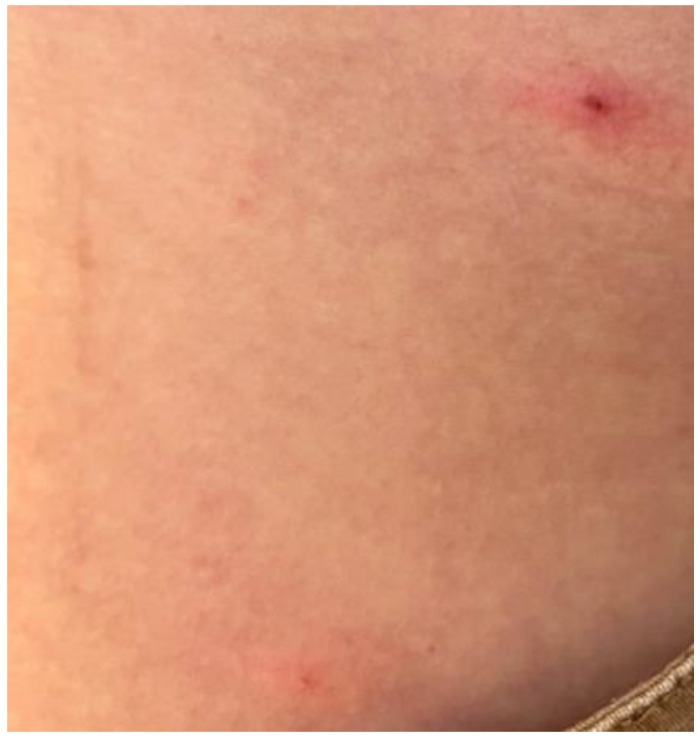
Clinical features related to some pustules on the trunk of the patient 1.

A 39-year-old Italian male came to our attention at the Complex Operative Unit of the University of Bari “Aldo Moro”, Apulia, Italy, after the failure of antiviral therapy (Aciclovir 400 mg twice a day) for a suspected herpetic infection prescribed by a different dermatologist. He reported a two-day history of fever, headache, and malaise, which were followed by the development of an erythematous plaque covered by vesicles on the chin, an oval ulcer with a white peripheral border and a central erosive area (Figure 1) on the lower lip, and three pustules on the arms and trunk (Figure 2). During the physical examination, cervical lymphadenopathy was also detected.

While recounting the personal medical history, the patient reported anonymous oral unprotected intercourse with a man at a rave party in Bologna, ten days before the initial symptoms. 

Due to the outbreak of the Mpox virus infection and the clinical presentation of the patient’s lesions, a skin swab of the exudative area of the chin was performed, and topical fusidic acid was prescribed to avoid bacterial superinfection.

The patient informed us that his boyfriend had a similar lesion, so we suggested that he, too, should come to our clinic. 

**Figure 3:**
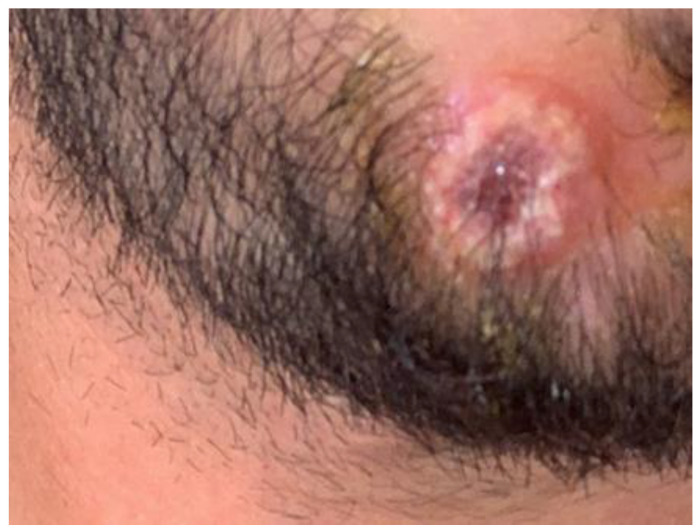
Clinical characteristics of patient 2 reported in this paper: note a single oval ulcerative lesion of the chin similar to the lesions of patient 1.

At clinical examination, a single oval ulcerative lesion of the chin (Figure 3) and cervical lymphadenopathy were observed, and patient 2 reported general symptoms that had arisen two days before the skin eruption. A skin swab of the lesion was performed, and we prescribed the same topical antibiotic cream to prevent superinfection. 

Serological and molecular research for sexually transmitted agents (syphilis, herpes, hepatitis B and C, and HIV) provided negative results. Clinical samples were processed at the Laboratory of Molecular Epidemiology and Public Health of Policlinico Hospital, Bari, Italy, which is the regional reference center for the diagnosis of the Mpox virus (MPXV). A real-time PCR assay was performed to detect the presence of MPXV in the clinical samples (skin lesions, whole blood, and nasopharyngeal swab), according to previously published methods [6]. Viral DNA was extracted via the Qiagen EZ1robot system (Qiagen, Italy, MI). All the clinical samples were positive for the presence of MPXV. Mpox virus infection was confirmed by skin swabs with a specific homemade real-time PCR.

It was decided not to subject the patients to any treatment as the clinical risk/benefit assessment did not suggest the use of any antiviral drug, but only supportive therapy.

After one month, our two patients presented protracted healing in the area with old lesions.

## 3. Discussion

Human Mpox (MPVX) is a zoonotic disease caused by the Monkeypox virus, a double-stranded DNA virus first isolated in monkeys in 1958 belonging to the Poxviridae family and to the Orthopoxvirus genus [7,8,9,10,11]. Small rodents are the virus’ natural reservoir, and both monkeys and humans are occasional hosts. It is endemic in the rural rainforests of Central and Western Africa, where it has been responsible for sporadic human cases and outbreaks since 1970. 

The biggest outbreak of Mpox disease in non-endemic countries started in May 2022 with the emergence of human cases of MPVX (H-MPVX). In Europe, most infections involve “men who have sex with men” (MSM), people with multiple sexual partners, and/or people who practice condomless sex. MPXV infection is not known to be a sexually transmitted disease (STI), but direct contact with broken skin during sex facilitates transmission. This could be the result of a spread in Europe that went undetected for a while, with human-to-human transmission due to close physical contact with infected asymptomatic or symptomatic adults. The disease has an incubation time ranging from 5 to 15 days and is characterized by a short prodromic phase (two–three days) with fever, chills, lymphadenopathy, malaise, headache, sore throat, myalgias, and gastrointestinal symptoms, followed by onset of the rash [11,12,13,14,15,16,17,18,19]. In patients with a complex exposure, the rash can precede the acme of febrile illness. 

As can be seen in our cases, lesions in patients in non-endemic countries are more topographically localized as compared to lesions in endemic areas and have a different distribution. Indeed, in the endemic area, following the prodrome, the rash begins on the face (95%) and soon spreads centrifugally to the palms, the soles of the feet (75%), oral mucous membranes (70%), genitals (30%), the conjunctiva, and the cornea (20%) [20]. Instead, in non-endemic areas, polymorphic lesions are common, mainly confined to the anogenital area, sometimes with rectal pain and penile edema [21]. In our cases, neither proctitis nor genital lesions were present, and only two days of unspecified general sickness were reported. This highlights the risk of underestimating these cases. Diagnosis must be confirmed by PCR testing of lesions or by the demonstration of IgM and IgG antibodies for MPVX in blood. The lesions may mirror the points of skin-to-skin sexual contact, with very few lesions appearing outside these anatomical sites, such as in our cases. The location of primary lesion sites matching those of sexual contact may lead us to consider a sexually transmissible infection [13,14,15,16,17,22]. Furthermore, colleagues from Spain reported a case of Monkeypox presenting with proctitis and disseminated maculopapular eruption, supporting the possible sexual origin [22]. Skin lesions follow a typical pattern of evolution, starting as macules and progressing into papules, vesicles, and pustules, which subsequently crust over and then desquamate [23]. Until the desquamation phase, when the crusting causes extreme itching, the lesions are unpleasant at all stages [24]. The lesions are often described as painful and later itchy. Scars with hyper/hypopigmentation could occur after scabs have fallen off. The development of the oral lesions should be similar to that of the skin lesions. The most prevalent oral scars are papulopustular rashes with scarring and crust development, manifesting as progressive, vesiculopustular, desquamating, and maculopapular necrotizing dermatitis in several forms. Oral blisters have a spherical shape and a crimson border, and after breaking off the roofs of the vesicle or pustule, ulceration with the pseudomembrane occurs. Some of these lesions develop umbilication, which can be seen as a dot on top of the lesion [25]. According to one study, mouth ulcers were present in nearly 23.5% of MPX patients [26].

Interestingly, lesions may occur in the same individuals at different stages. These oral symptoms have never before been described in patients in the endemic region [27]. In a study carried out by Thornhill et al. in 2022, 26 people demonstrated oropharyngeal symptoms such as oral or tonsillar lesions, odynophagia, epiglottitis, and pharyngitis as the first symptoms [28]. Moreover, small, fragile blisters on the mucosal membrane, painful aphthous ulcers, multiple scattered lesions inside the mouth and on the lips, facial rashes and severe pain, petechial lesions on the hard palate, and temporomandibular joint stiffness are indicative of subclinical immunosuppression associated with a Mpox infection [29]. Severe manifestations of infection include encephalitis, secondary skin infection, pneumonia, tonsillitis, hemorrhagic pustules, pharyngitis, edema of the eyelids, and ocular disease leading to loss of vision [29].

As for our two cases, the prognosis was excellent, with spontaneous resolution in one–two weeks. 

However, severe outcomes have been described in children, pregnant women, and immunocompromised hosts, so these patients deserve special attention [17].

## 4. Conclusions

These case series highlight the difficulties encountered by clinicians facing patients with MPXV infection during the ongoing outbreak outside Africa and points out the importance of dermatological counseling for patients with atypical skin lesions and clinical history.

Finally, the present report suggests that the transmission of Mpox through oral or anal sex is probable, hence it is essential to gain a better understanding of the epidemiology of the Mpox virus to prepare clinicians and public health specialists ready for the likely spread of this disease in non-endemic countries.

## Data Availability

Not applicable.
